# Stakeholder analysis of childhood obesity prevention policies in Iran

**DOI:** 10.1186/s13690-021-00557-9

**Published:** 2021-03-17

**Authors:** Shahnaz Taghizadeh, Rahim Khodayari Zarnag, Mahdieh Abbasalizad Farhangi

**Affiliations:** 1 Student Research Committee, Faculty of Nutrition, Tabriz University of Medical Sciences, Tabriz, Iran; 2 Department of Health Policy and Management, School of Management and Medical Informatics, Tabriz University of Medical Sciences, Tabriz, Iran; 3 Department of Community Nutrition, Nutrition Faculty, Tabriz University of Medical Sciences, POBOX: 14711, Attar Nishabouri St., Tabriz, 5166614711 Iran

**Keywords:** Stakeholder analysis, Childhood obesity, Prevention, Policy

## Abstract

**Background:**

Childhood obesity is one of the most serious health challenges and risk factors for various diseases. International health organizations, such as the World Health Organization (WHO), recommend more stakeholder engagement to discuss this health problem. Therefore, this study aims to identify and analyze the stakeholders of childhood obesity prevention (COP) policy making process in Iran.

**Methods:**

In this study, semi-structured interviews were conducted with 24 Iranian stakeholders in the field of COP from February 2019 to May 2020. PolicyMaker V.4 and MAXQDA V.10 software were used for the analysis of stakeholders.

**Results:**

Out of 24 interviewed stakeholders, 17 were active and seven were inactive. The most powerful and supportive stakeholders were the Ministry of Health and Medical Education (MoHME) and the national and regional Offices of Community Nutrition Improvement. Although stakeholders like the Ministry of Sports and Youth, the municipalities, the Secretariat of the Health and Food Safety, the Islamic Republic of Iran Broadcasting (IRIB), and Student Organization were among powerful bodies, they showed the least support for COP policies. The remaining stakeholders showed medium or low support and power in the policy making process.

**Conclusions:**

Disconnect between stakeholders, less attention to prevention strategies, and high advertising of unhealthy foods were among the main challenges facing the COP policies in Iran and worldwide. Insufficient financial resources, little attention to the young people under 18, non-aligned policies of the Student Organization and the IRIB, and insufficient support of COP policies were among the key challenges to be handled.

## Background

Childhood obesity is one of the most serious health challenges and risk factors for cardiovascular, metabolic, gastrointestinal, and other diseases [1]. According to the report by the World Health Organization (WHO), 40 million children under the age of 5 were overweight or obese in 2018 and over 340 million children and adolescents aged 5–19 were overweight or obese in 2016 [2]. Meanwhile, the rate of childhood obesity in developing countries was 30% higher than in developed countries [3]. The results of a surveillance study entitled “Childhood and Adolescence Surveillance and Prevention of Adult Non-communicable Disease” (CASPIAN-V) conducted on 14,118 subjects aged 7–18 years in 30 provinces of Iran showed that the prevalence of overweight and obesity was 9.4 and 11.4%, respectively [4]. Several previous studies showed that the upward trend of childhood obesity will impose high costs on the health system and will have irreparable consequences for the individuals and society [5, 6]. Many factors such as consuming more energetic foods or lack of physical activity (PA) can affect the prevalence of obesity in this age group [7, 8]. In a meta-analysis, Norris et al. demonstrated that PA in schools significantly improved students’ academic performance and had a positive effect on health [9]. Moreover, the CASPIAN-V study carried out in 2014 showed that 58% of children and adolescents had a low level of PA and 53.6% of them had a low or moderate adherence to healthy nutritional behaviors [10].

Accordingly, the WHO advises governments to take population-based strategies to combat obesity in children and adolescents [11]. Due to the increasing prevalence of childhood inactivity among different countries of the world, the WHO developed a plan in 2018 to increase PA among young people under 18. Therefore, different organizations and stakeholders should make appropriate policies in this respect. Studies show that, similar to other countries [12], there are conflicts of interest among stakeholders in the healthcare system and that health priorities are not prioritized properly in Iran [13]. Adhikari et al. evaluated the Malawian health system stakeholders and showed that having problems in coordinating between stakeholders would create many problems for health system policies [14]. Regarding the dominance of a curative approach over preventive approach in Iran [13], it seems that the analysis of stakeholders is important for future policies related to childhood obesity prevention (COP). In this regard, the results of a study in Brunei to check the strengths and weaknesses of COP policies through semi-structured interviews with the government and school stakeholders indicated that most stakeholders blamed parents for the problem of childhood obesity. This individualization of the problem from the perspective of stakeholders was identified as a barrier to the planning and implementation of relevant COP interventions [15]. As far as the researchers investigated, so far, no comprehensive study has been carried out to identify the role of key stakeholders and actors related to COP in Iran. Therefore, this study aims to identify and evaluate the stakeholders of COP policy making process in Iran.

## Materials and methods

In-depth, semi-structured, one-to-one interviews were used to explore the perceptions of stakeholders of the COP policies in Iran. This qualitative stakeholder analysis is part of a larger qualitative exploratory study entitled “A Future Study and Policy Analysis of the Prevention of Obesity in Children and Adolescents in Iran and Providing Policy Options”. The study setting was on the organizations and institutions involved in Iran’s COP policies. Policy, as used in this study, refers to any national, regional, or local program, law, regulation, or rule related to COP [16]. Key informants were identified as targeted sampling method; over time, using snowball sampling method, interviews were conducted up to the data saturation. Finally, 24 key informants were interviewed. The aims of the study were provided to all participants and a consent was obtained from them. To make participants at ease when answering the questions, the anonymity of all data was assured.

PolicyMaker software V.4 was used in the analysis process; this software has been designed by Harvard University and organizes stakeholder information, provides guidance on strategies to deal with the stakeholders, and creates effective visuals for presenting the information to policymakers [16]. Following the recommendations by WHO in the analysis of stakeholders, the MAXQDA V.10 software and Kammi Schmeer model [16] were used for designing and implementing the study. As can be seen in Table 1, there were eight stages in current study.

## Results

In this study, 24 informants were interviewed and 3 stakeholders (General Directorate of Standards, Regional Broadcasting Organization, and Department of Economic Affairs and Finance) rejected our invitation to participate in the interviews (response rate = 88.8%). The general characteristics of the stakeholders were as follows: there were eight policymakers and 16 executive managers (12 women and 12 men). The average work experience of stakeholders was 14.65 years and their average age was 47.86 years old. The results were categorized into nine sections and stakeholders’ analysis findings were presented based on the results of the document analysis and interviews. The characteristics of participants and their positions are shown in Table 2. The key stakeholders were analyzed according to the stakeholder analysis guidelines. PolicyMaker software (version 4) was also used to assess stakeholder characteristics more accurately [16].

**Table 1:** The process of stakeholder’s analysis in childhood obesity prevention policies

stage	Name of stage	Processes of each stage
First stage	Planning the process	• Formation three sessions working group • Definition the purpose of the analysis, determining the approximate timeline, how to conduct the interview process, determining the goals and how to use the results during the sessions • Review relevant website, documents and articles
Second stage	Selecting and defining a policy	• Consulted a number of policy makers (to define child and adolescent obesity prevention policies) • Coordination meeting with the research team
Third stage	Identifying key stakeholders	• Develop the priority stakeholders list with possible to be added in the next steps as snowballs
Forth stage	Adapting the tools	• Developed a standard questionnaire (open-end) for interviewing stakeholders • Review existing information from possible sources (e.g. newspapers, institutional reports and publications, websites and other available documents) • Develop the interview protocol: (conducted as pretest the interview guideline with 4 nonpriority stakeholders to determine the comfortable, understands the questions and the time line of interviews)
Fifth stage	Collecting and recording the information	• Conducting interviews (after previous coordination) • Created a separate electronic file for each stakeholder • Enter the stakeholder’s answers for each question into the computer (immediately following the interview)
Sixth stage	Filling in the stakeholder table	• Transferred the interview responses to the table (using the completed interview guidelines and electronic files for each of stakeholders)
Seventh stage	Analyzing the stakeholder table	• Analyze the findings in the results of interviews using policy maker and MAXQDA10 software. • Divide the stakeholders into nine groups which are shown in the form of a matrix in Table 3.
Eighth stage	Using the information	• The results of the analysis were collected so that it could be used in various areas such as action plans to increase support for a reform policy, policymakers and policy providers

**Table 2:** The characteristics of participations in the study

Player name	Participant’s No	Status of stakeholder (A or P)	Level	Sector	Position	Power	Interest	incentives	Awareness of policy	Related role
Food and Drug Organization	P1	P*	National	Governmental	Medium Support	High	Medium	Non-financial	High	Policy making for increase the level of safety of food and beverages
Islamic Republic of Iran Broadcasting (IRIB))	P2	P	National	Media	Low Support	High	Low	financial	Medium	Producing educational and informational programs as well as harmful food ads
The Office of Community Nutrition Improvement	P3	P	National	Governmental	High Support	High	High	Non-financial	High	Mainly responsible for the COP: Policy making, design and implementation of nutritional interventions
Health Deputy of MoHME***	P4	P	National	Governmental	High Support	High	High	Non-financial	High	Policy making for health service system in Iran
Department of education (Professor of university of medical sciences of MoHME(	P5	P	National	Governmental	High Support	High	High	Non-financial	High	Cooperation with the MoHME and WHO in promoting Iran ECHO, Responsible for implementing Iran ECHO pilot program in Isfahan (one of the cities in Iran)
Ministry of Education	P6	P	National	Governmental	Medium Support	High	Medium	Non-financial	High	Collaborate with the MoHME in develop or implement the nutrition education, school foods or PA^a^ policies in schools
School Health Department of MoHME	P7	P	National	Governmental	Medium Support	Medium	Medium	Non-financial	Medium	Policy making for nutrition education, providing healthy food, as well as training and doing PA in schools
Welfare Organization	P8	P	National	Governmental	Medium Support	Medium	Medium	Non-financial	Medium	Policy making for nutrition education to children and parents and adherence to the principles of healthy nutrition in kindergartens and childcare centers
Children and Adolescents’ Intellectual Development	P9	A**	Regional	Governmental	Medium Support	Medium	Low	Non-financial	Low	Participation in healthy nutrition education for children and adolescents
The office of Community Nutrition Improvement department of the Health Center	P10	P	Regional	Governmental	High Support	High	High	Non-financial	High	Policy making, design and implementation of nutritional interventions in the provincial level
Executive manager in the Ministry of Education	P11	P	Regional	Governmental	Medium Support	Medium	Low	Non-financial	Medium	Collaborate in implementation of the nutrition education, and PA policies in schools
Food and Drug Office	P12	p	Regional	Governmental	Medium Support	Medium	Medium	Non-financial	Low	Increase the level of safety of food and beverages
Imam Khomeini Relief Committee	P13	A	Regional	Non-Governmental	Low Support	Medium	Low	Non-financial	Low	Providing healthy food items for financial trouble families as well as education healthy lifestyle in family training sessions
Ministry of Sports and Youth	P14	A	Regional	Governmental	Low Support	High	Low	financial	Low	Providing appropriate facilities for exercise in childhood
Municipality	P15	p	Regional	Non-Governmental	Low Support	High	Medium	Non-financial	Medium	Providing sports equipment and spaces for children and adolescents
Population and Family Health Department	P16	P	Regional	Governmental	Medium Support	Medium	Medium	Non-financial	Medium	Collaborate on primary health care and screening for overweight and obese children and adolescents in health centers
School Health Department of the Province’s Health Center	P17	P	Regional	Governmental	Medium Support	Medium	Medium	Non-financial	High	Coordinate with schools to provide nutrition and PA training and screening overweight and obese children and adolescents
Student Organization	P18	P	Regional	NGO	Low Support	High	Low	financial	Low	Collaborate on providing school buffet snakes
Department of education) Professor of university of medical sciences of MoHME(	P19	P	Regional	Professional	Medium Support	Medium	Medium	Non-financial	Medium	Collaborate in developing policies to prevent childhood obesity by nutrition education in the university
Welfare Organization	P20	A	Regional	Governmental	Low Support	Low	Low	Non-financial	Low	Collaborate on nutrition education for children and parents in kindergartens and observing the principles of healthy nutrition in kindergarten food and child care centers
Secretariat of the Health and Food Safety	P21	A	Regional	Governmental	Low Support	High	Low	Non-financial	Medium	Establish better coordination and communication between stakeholders and organizations
Health care providers of MoHME ***	P22	P	Regional	Governmental	Medium Support	Medium	Medium	Non-financial	Medium	Collaborate on primary health care, nutrition education and PA advice screening for overweight and obese children and adolescents in health centers
Islamic Development Organization	P23	A	Regional	Governmental	Low Support	Medium	Medium	Non-financial	Low	Advertising of nutrition and PA in the community
Non-communicable diseases Department of the Province’s Health Center	P24	A	Regional	Governmental	Medium Support	Medium	Low	Non-financial	Medium	Collaborate on development of guidelines of non-communicable diseases prevention
Social Security Insurance Agency	NP****	A	National	Governmental	–	–	–	–	–	Coverage of nutrition counseling services for nutrition counselors and specialists
Ministry of Economic Affairs and Finance	NP	A	National	Governmental	–	–	–	–	–	Allocation of taxes for imports as well as Value-added tax (VAT on unhealthy foods as well as subsidy allocation for healthy foods
Islamic Consultative Assembly (Iranian Parliament	NP	A	National	Governmental	–	–	–	–	–	Upstream legislation and resource allocation for the executive organizations

* P: Present** A: Absent, *** Ministry of Health and Medical Education, **** Not participating in the study

^a^ physical activity

**Table 3:** Estimation of position and power of childhood obesity prevention policies in Iran (in 2020)

POWER				
POSITION		Low	Medium	High
	High supportive	-	-	✓ The Office of Community Nutrition Improvement (N*) ✓ The Office of Community Nutrition Improvement Department of the Health Center (R**) ✓ Health deputy of MoHME ***(N*)
	Medium supportive	-	✓ School Health Department of MoHME (N*) ✓ Welfare Organization (N*) ✓ Children and Adolescents Intellectual Development (R**) ✓ Population and Family Health Department (R**) ✓ School Health Department of Health Center (R**) ✓ Department of education, university of medical sciences ^a^ (R**) ✓ Health care providers of MoHME (R**) ✓ Executive manager in the Ministry of Education (R**) ✓ Food and Drug Office (R**)	✓ Ministry of Education (N*) ✓ Food and Drug Organization (N*)
	Low supportive	✓ Welfare Organization (R**)	✓ Imam Khomeini Relief Committee (R**) ✓ Islamic Development Organization (R**) ✓ Non-communicable diseases department of the province's Health Center (R**)	✓ Ministry of Sports and Youth (R**) ✓ Secretariat of the Health and Food Safety ✓ Islamic Republic of Iran Broadcasting (IRIB)) (N*) ✓ Student Organization (R**) ✓ Municipality (R**)

*N: National, **R: Regional, *** Ministry of Health and Medical Education, ^a^ this stakeholder is university professor

### Identification of stakeholders

#### Introducing some organizations of the stakeholders

In this section, a brief description of some organizations that may be unfamiliar to the readers is presented; then the results of this study are discussed.

According to the latest information, Iran is divided into 31 provinces and each province into several cities. There are 1240 Municipalities in Iran. Each city has a main municipality and large cities have several regional municipalities. The municipalities are a public, non-governmental, local, and self-sufficient organizations, and their major source of income is through collecting taxes and tolls from the citizens. Cities with more than 200,000 people are divided into different areas (regional) [17, 18]. The municipalities are responsible for facilitating safe and suitable places for PA in parks or in the cities, which can be effective in obesity prevention [19].

The Welfare Organization of Iran is a government agency and sector of the Ministry of Welfare and Social Security of Iran. Part of the responsibilities of this organization is to license and manage kindergartens for people under the age of 6 in the country. In addition, one of the duties of this organization is to teach healthy lifestyles to children and parents. Since 2007, a warm meal per day is provided to the students at rural kindergartens, which is implemented by the Welfare Organization; and nutrition education is one of the goals of this program [20].

The Student Organization is a public and non-governmental organization affiliated to the Ministry of Education (MoE); however, it is not a subset of the MoE. Strengthening social education and planning for students’ leisure times is one of the most important goals of this organization. One of the tasks of this organization is to monitor the quantity and quality of food products or snacks sold in school canteens (buffets). Buffets are more like a kiosk, where students buy snacks such as cakes and biscuits. It is necessary to mention that, the buffets are available in both public and private schools; but in most private schools, some warm foods such as pasta, soup, etc. are also provided. The distribution of these warm foods is not the responsibility of the Student Organization and the schools are responsible for preparing and distributing these foods.

#### The present and absent stakeholders

Given the multifaceted nature of COP policies, many stakeholders at the levels of prevention and support can involve in related policies and programs. Based on the existing documents and supplementary views of the key informants, the findings of current study showed that several stakeholders do not have significant and clear roles in childhood and adolescent obesity prevention programs despite their high potentials in this respect. We categorized these stakeholders as absent (A), and the rest of the stakeholders that were somehow involved in the policies, though to a small extent, as present (P) stakeholders (Table 2).

### Characteristics of the stakeholders

As Table 2 shows, the characteristics of the stakeholders are presented in eight areas. The level of stakeholders is divided into two national or regional levels. Among the stakeholders interviewed, eight stakeholders were national and 16 were regional. Given that having the power to participate and support the policies is very important, we examined the level of support from stakeholders in power. Table 3 shows the impact of power and position of stakeholders in the process of policymaking; we have also described the position of stakeholders with high power in detail. It should be noted that in the PolicyMaker software, the level of support for the policies refers to the stakeholders’ approach, perspective, and position as well as using their power and facilities in this respect.

#### High supportive stakeholders with high power

The most powerful and supportive stakeholders were the Health Deputy of MoHME and the national (policymaking) and regional (policy implementation) Offices of Community Nutrition Improvement. In 2014, Iran’s healthcare system took a major step in preventing non-communicable diseases by creating a new system for providing services called Health Transformation Plan. One interviewee from the Office of Community Nutrition Improvement explained that:

“*After the health transformation plan, two important things happened that are unique: one is the use of nutrition experts and the other is that the programs were integrated with the view of preventing non-communicable diseases, obesity, and overweight*” (P3).

Since 2016, the Iran ECHO (IRAN-Ending Childhood Obesity) program, which is a proposal of the WHO to end obesity in the world, has been implemented in some provinces of Iran.

#### Medium supportive stakeholders with high power

The medium supportive stakeholders with high power included MoE and Food and Drug Administration of the MoHME. There are two main challenges for MoE stakeholders. Firstly, it seems that despite the high power for practical school policymaking, school-based communication policies are not properly implemented by executive managers. In addition, MoE stakeholders believe that their organization is not directly responsible for preventing obesity in children and adolescents*:*

*“School buffets should be constantly inspected and controlled by education administrators, but it is not fulfilled appropriately”* (P17).

Secondly, the follow-up at the executive level in schools is insufficient and inefficient. Physical education is not effectively implemented in schools that do not have a sports hall, especially in snowy or rainy days. In addition, some health-related policies in the MoE are often implemented inappropriately due to lack of financial resources and staff. Sport centers exist in some large public schools, that are designed to fill the leisure time of interested students, but due to poor management and the need for students to paying a fee, students do not have much desire to participate in these centers. Nutrition and health education textbook is intended for the high school (12th grade). However, the time of this course and physical education course is usually devoted to other courses required in the university entrance exam, and unfortunately, policymakers have not developed effective policies in this regard. Another problem in this organization is the inadequacy of sports equipment in schools as well as, physical education teachers in primary schools.

The Food and Drug Administration of the MoHME has designed plans to prevent obesity in the community including: 10% reduction in the sugar content of carbonated beverages since 2015, reduction of trans fatty acids in edible oils from 58% in the early 1980s to 5% in confectionery oils and 2% in edible oils in 2018 [21], and installation of nutrition signs and nutrition labels on the produced foods. One of the stakeholders of this organization said:

*“Encouraging the production of health-oriented goods is another existing policy for society to encourage producer, so that the product has a minimum of calories” (P1).*

However, some stakeholders believe that there is not enough monitoring by the Food and Drug Organization regarding the production of unhealthy foods.

“*The student buys unhealthy food such as chips, puffs, etc. outside of school; and it is the economic abuse in the producer that there is no hesitation in producing unhealthy food*” (P18).

#### Low supportive stakeholders with high power

These stakeholders are mainly the Ministry of Sports and Youth, the Municipalities, the Secretariat of the Health and Food Safety, the IRIB, and the Student Organization. In the Sports and Youth Organization, less attention has been paid to the young group under 18. Accordingly, one of the officials of this organization expressed:

“*We have sporting programs for all ages in public, but such programs are very rarely arranged for the children and teenagers age groups”* (P14).

This stakeholder also cited the inadequate support of the Sports and Youth Organization in this regard:

“*We do not have directions and instructions on the prevention of obesity in children and adolescents; hence, little work can be done in this age group*” (P14).

The Municipalities have high power in this policy, and their attention to health issues has increased by creating a unit called “citizenship health” and recruitment of nutrition experts in all municipalities of the country in the last decade. Since Municipalities are public and non-governmental organizations, they have a great power in policymaking, though some challenges restrict this power. One of the stakeholders of this organization said:

“W*e are in charge of urban management and we are obliged to inform the people and create health houses, hold health-related conferences and meetings, and increase PA and healthy eating. We have tried to do so; but at the moment, we do not have a special program for children or adolescents*” (P15).

Despite the fact that Municipalities have programs for reducing obesity among adults and their employees interviewed in this study showed a great interest and desire to participate, however, childhood obesity has not been considered specifically. On the other hand, the COP has been ignored in the policies of this organization; insufficient PA facilities and equipment for this age group in the cities, is one of the signs of this claim.

An employee of the Secretariat of the Health and Food Safety expressed that:

*“This office collects reports on the health-related activities of various organizations, and links medical sciences with other organizations such as the governor's office, etc. In the case of children, nothing has been done absolutely”* (P21).

Although the Secretariat of the Health and Food Safety is a powerful organization in COP policies, it is completely inactive in preventing childhood obesity*.*

Regarding the role of IRIB, this organization had important guidelines on health, nutrition, and PA in the form of films and educational programs, but the target group of most of these programs were adults. On the other hand, unhealthy and obesogenic food commercials often target children and adolescents groups; but this organization is reluctant to stop such commercials due to the high amounts of money received from the manufacturers of these unhealthy foods. In this regard, the stakeholders believed that this organization does not play an effective role in preventing childhood obesity, and even in some cases, it blocks the implementation of these policies.

“*One of the major problems is that stakeholders such as radio and television )IRIB) do not collaborate appropriately*” (P 11).

The Student Organization is not a subset of the MoE, and most stakeholders complain about the lack of cooperation and coordination between them, or the health system regarding the snacks provided in school buffets. On the other hand, since this organization is also responsible for organizing programs for students’ leisure time, it should provide nutritional, educational, and PA-related programs for students based on the structure of Iran; but this has not been fulfilled efficiently.

### Stakeholders’ participation rate

**Fig. 1:**
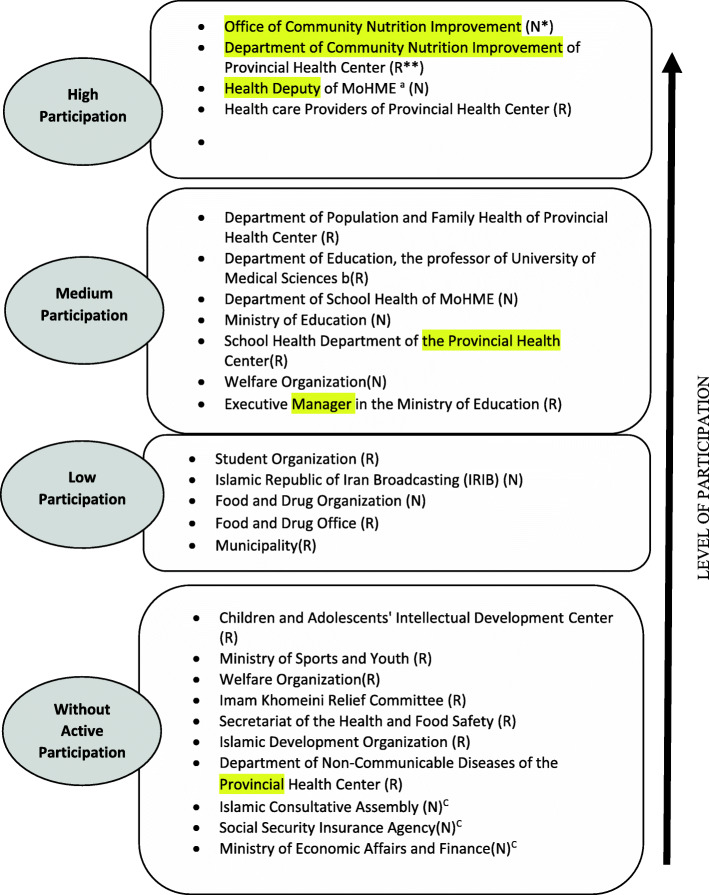
Level of stakeholder’s participation, * National, ^a^ Ministry of Health and Medical Education, ^b^ This stakeholder is university professor, ** Regional, ^C^ These stakeholders did not participate in the interview, but their non-participation was obtained from the documents and interview results of other stakeholders

The participation of the stakeholders refers to the issues of participants, level of involvement, intensity, timing and goal of participation, in addition to using the available facilities for this policy [22]. Given the importance of stakeholder participation in this policy, the participation rate was divided into four groups as high, medium, low, and without active participation (Fig. 1).

#### High participation

The Health Deputy of MoHME and the Office of Community Nutrition Improvement, as well as healthcare providers of Provincial Health Center have a strong interest in participating. The MoHME is involved in policymaking by providing guidelines such as communicating instructions to the centers under its auspices to provide nutrition and healthy lifestyle training to clients, as well as working with other organizations and stakeholders on overweight and obesity screening and lifestyle education. The MoHME and its Office of Community Nutrition Improvement were identified as the most important organizations responsible for nutrition issues in Iran. It seemed that these organizations participated through recruitment of nutrition experts and increasing the number of healthcare providers in health centers in recent years. Healthcare providers, had a high participation; but they complained about the multiplicity of tasks assigned to them. They have to do primary healthcare, anthropometry, training nutrition and healthy lifestyle for all age groups, and especially prenatal care, childhood care, as well as management of most non-communicable diseases.

#### Medium participation

Some other departments of the MoHME, such as Department of Population and Family Health of Provincial Health Center, Department of School Health of MoHME, and a professor at the University of Medical Sciences who participated in the interview, had medium participation. The level of participation of university professors is limited to the education of health and nutrition students; these people will be responsible for the COP policymaking and implementation in the future. Therefore, their level of participation is high, though the intensity and number of their participants in this field is low. Department of Population and Family Health, in addition to exclusive breastfeeding training, provides brief training on nutrition and physical activity. These stakeholders complained that the COP-related policies are not fully implemented due to the multiplicity of primary healthcare processes, their high workload, and lack of enough time and staff. Nevertheless, the large number of staff involved and their time spent on anthropometry, nutrition and lifestyle education, and low level and intensity of participation in health centers caused these stakeholders to be placed in the group with medium participation.

The Department of School Health is involved as a liaison between schools and health centers; it also holds some health conferences on PA and nutrition. However, the focus of this department is on such processes as prevention of pediculosis and improving oral health, and not COP policies. Although the number of participants in this organization is not large and they have different goals in establishing this relationship, the participation level and intensity of participation related to COP policies is high.

MoE in the policymaking level, as well as executive level in the MoE, despite having high potentials, showed a moderate interest in participating; it seems that they considered COP policies as irrelevant to their scope. Their role is in nutrition education in some grades at school, and also PA education in all grades. However, the results showed that the PA education and implementation is inappropriate. The MoE stakeholders believed that it is difficult to implement some of the educational and executive policies in classrooms. These stakeholders were interested in implementing the policies of their own organizations and they were reluctant to cooperate with other organizations, such as the MoHME programs.

The Welfare Organization was another stakeholder which was reluctant to implement the related policies. Despite the support and cooperation at the policymaking level in this organization, there were many problems at the implementation level. The most important reason identified for these problems in the current study was the lack of financial resources. In this regard, one of the executive managers of this organization announced that:

*“First of all, we do not have the budget to implement these policies. Since we are unable to provide enough budget for kindergarten teachers because of the economic problems, it is impossible to expect providing adequate education in kindergarten for children and parents as well as providing quality food and healthy diet for children in rural kindergartens”* (P20).

#### Low participation

As mentioned earlier, Municipalities can have an indirect impact on COP by holding healthy lifestyle and nutrition education sessions for family adults. Another organization that has a great potential for policymaking in this field is the Ministry of Sports and Youth; however, their policies are mainly focused on youth and adults and not on the people in the age range of 0–18 years old. They believe that a student’s PA should be done in schools and they have no role in this process. Apparently, the Student Organization is involved in implementing COP policies; however, according to most stakeholders in the MoE and MoHME, because this organization is not a subset of MoE, the stakeholders of this organization contract with some obesogenic food producers for financial gain in order to sell their products in student buffets.

Despite the fact that national broadcasting organizations have the strongest potentials to increase health awareness in any society, IRIB was one of the most controversial organizations in COP policies due to the getting huge budgets from the producers of unhealthy foods for advertising their products. In this regard, one of the MoE stakeholders claimed that:

“... *but the commercials related to unhealthy foods destroy our efforts (P7)*”.

Stakeholders believed that the Food and Drug Organization, despite having great potential to participate in COP policies, has not been very active in this area, except for a few cases of reducing sugar and fat in some food products and installation of nutrition signs and labels on produced foods. Therefore, according to other stakeholders, this organization could have been primarily responsible for producing healthier, low-fat, and low-energy foods, which has not been fulfilled. Other stakeholders who have not taken any initiative in this area or have not collaborated with the health system in preventing childhood obesity are classified as stakeholders without active participation (Fig. 1).

## Discussion

In general, the economic, political, social and cultural conditions of any society have potential effects on the health [23]. Considering that the MoHME is the main responsible body for health in Iran, different approaches have been adopted to prevent chronic non-communicable diseases such as childhood obesity. There have been many changes in recent decades in the Iranian health system; for example, the family physician program started in 2005 [24], and the health transformation plan started in 2014 [25]. Despite improvements in the health system [26], there were adverse effects on overall healthcare policies such as non-communicable disease prevention. Pindus et al. in their study demonstrated that changes in the health system, in addition to having benefits, can also have some adverse effects [27]. Regarding the multidimensional nature of childhood obesity [28], the prevention of obesity in this age group requires a wide inter-sectoral collaboration among stakeholders [29]. The results of the current study showed that coordination between the stakeholders is weak, which can lead to a major problem in policymaking and implementation. A similar result was reported by Adhikari et al., in which the analysis of stakeholders showed that having problems in coordinating between stakeholders would create many problems for health system policies [14]. Another issue is the existence of a top-down approach in the Iranian health system [30], which is not unique to Iran; and this is one of the challenges of the health systems [31]. In a top-down policymaking system, which policymakers do not engage with executive and local stakeholders, in most cases, the desired result is not achieved and may lead to the destruction of the initiatives and policies developed [32].

More focus on curative approach than prevention one in the Iranian health system is another challenge in the policies adopted by the stakeholders. This result was similar to the findings of the study by Doshmangir et al., in which stakeholders believed that the Iranian government was more focused on curative approaches, while, less attention was given to public health and preventive interventions [13]. The results of our study were also similar to the McCollum et al. study, that showed that in Kenya and Indonesia, curative approaches have overcome prevention that have adverse effects on the health system [33]. However, studies have shown that COP is more effective than curative approaches and prevention is the key to success for obesity control [34]. Another issue is that most stakeholders expected that the MoHME would be responsible for policymaking and implementation of COP, but as studies showed, effective interventions in this area require the participation of stakeholders in various sectors. Some of these various sectors stakeholders can include the agriculture section, by allocating subsidies to farmers to produce fruits and vegetables, the education section, by widely using the healthy lifestyle training in the schools and mass media, the existence of appropriate policies in the city for doing PA such as cycling for children and adolescents or creating safe crossings for walking [30, 35], and fundamental changes in lifestyle [36]. In 2015, the study by Abu-Omar et al. [37] showed that PA-friendly environments are one of the important facilitators for COP. The challenges such as inappropriate programs in the Sports and Youth Organization for this age group, inadequacy of sports equipment in the schools and physical education teachers in primary schools, and inadequate time of physical education courses (less than 1.5 h per week) were the major problems in policymaking for the PA of children and adolescents. Although, many studies have shown that school-based COP policies have shown the best results [38–40], the approved PA hours for schools in Iran is 2 h/week, which is equivalent to 7.5% of the total curriculum, which is low compared to most countries where they have an average of 2.3 h/week of PA (equivalent to 9% of the total curriculum) [41]. It should be noted that the amount of PA in schools is actually less than 2 h per week. Another obstacle to PA in schools is the small size of schoolyards, which is often seen in private schools. This result is similar to the findings of Moges et al., in which about one-half of private schools in Addis Ababa, Ethiopia, had inadequate play area in the private schools, which was significantly associated with overweight/obesity [42].

Some non-governmental organizations (NGOs) involved in this policy, such as the Student Organization, despite their high potential, did not show adequate participation in advancing COP policies. Some studies have showed that most NGO activities in Iran have been performed in the area of providing services, and few of them are related to policymakers [30]. The main NGO in this field is the Student Organization, which unfortunately, plays a negative role in advancing COP policies in Iran. Although this NGO is the principal trustee of school buffets, it does not have proper policies to control school buffets and contract with certain food manufacturers whose products are obesogenic. Similar to the study by Louise et al. [43], fast food and soft drink taxing was regarded as a suitable intervention by some stakeholders. Unhealthy food advertising is one of the most important contextual challenges for which appropriate policies have not been developed or implemented. According to the Institute for Fiscal Studies, half of television advertisements for food and drink seen by children are related to products with high levels of fat, salt, or sugar. Unhealthy food advertising has been shown to be one of the obstacles to the implementation of COP policies in most studies [44–46].

Accordingly, the Iranian parliament can contribute in related legislation such as taxing unhealthy and obesogenic foods, allocating subsidies for healthy foods, and allocating funds for the free distribution of healthy foods in schools; but most stakeholders complained about the government’s insufficient support for this policy. However, in a study in the United Kingdom entitled “Healthy Weight, Healthy Lives (HWHL)” to prevent obesity, a stakeholder analysis of the program showed that success in such programs was due to government stakeholders direct participation and support as well as timely financing by relevant stakeholders [29]. Another French study called, ‘Ensemble Prévenons l’ObésitéDes Enfants’ (EPODE), showed that by involving different stakeholders in various fields, significant achievements were made in the implementation of these policies.

## Conclusion

According to our results, the following were among the main challenges of COP policies in Iran, some of which can be generalized to other countries: disconnect between COP policies, implementation, and stakeholders; less attention to prevention approach than curative one; and high advertising of unhealthy foods. Other challenges include insufficient budget allocation to implement COP policies, not classifying age groups in the policymaking, and less attention to the age group under 18 years. Future issues such as delegating student buffet authority to a non-governmental organization and the non-use of existing powers and resources by most stakeholders, demonstrate the fundamental need for advocacy in this area.

## Data Availability

The datasets used and/or analyzed during the current study are available from the corresponding author on reasonable request.

## Abbreviations

CASPIAN: Childhood and Adolescence Surveillance and Prevention of Adult Non-Communicable Disease
COP: Childhood Obesity Prevention
ECHO: Ending Childhood Obesity
HWHL: Healthy Weight, Healthy Lives
IRIB: Islamic Republic of Iran Broadcasting
MoE: Ministry of Education
MoHME: Ministry of Health and Medical Education
WHO: World Health Organization
